# Spatial Access to Vaccines for Children Providers in South Carolina: Implications for HPV Vaccination

**DOI:** 10.5888/pcd17.200300

**Published:** 2020-12-24

**Authors:** Radhika Ranganathan, Whitney E. Zahnd, Sayward E. Harrison, Heather M. Brandt, Swann Arp Adams, Jan M. Eberth

**Affiliations:** 1 Rural and Minority Health Research Center, Arnold School of Public Health, University of South Carolina, Columbia, South Carolina; 2 Big Data Health Science Center, Arnold School of Public Health, University of South Carolina, Columbia, South Carolina; 3 Department of Psychology, College of Arts and Sciences, University of South Carolina, Columbia, South Carolina; 4 South Carolina SmartState Center for Healthcare Quality, Arnold School of Public Health, University of South Carolina, Columbia, South Carolina; 5 Department of Health Promotion, Education and Behavior, Arnold School of Public Health, University of South Carolina, Columbia, South Carolina; 6 Cancer Prevention and Control Program, University of South Carolina, Columbia, South Carolina; 7 College of Nursing, University of South Carolina, Columbia, South Carolina; 8 Department of Epidemiology and Biostatistics, Arnold School of Public Health, University of South Carolina, Columbia, South Carolina

**Figure:**
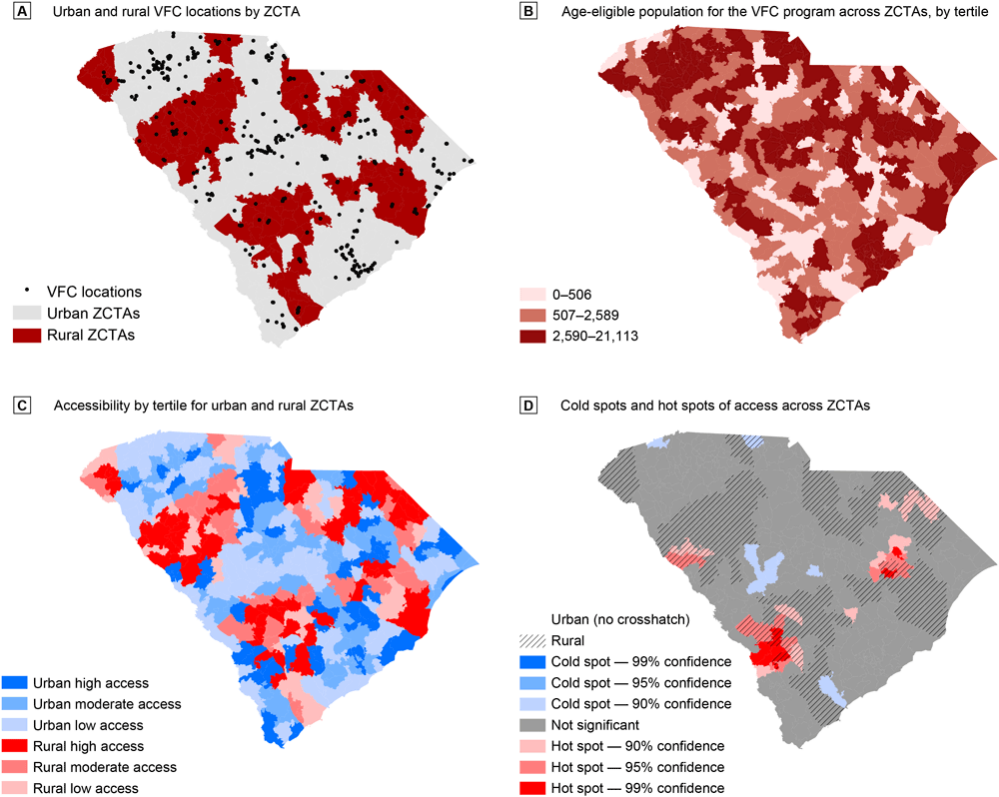
Panel A shows the location of VFC providers by rural and urban Zip Code Tabulation Areas (ZCTAs). Panel B shows the age-eligible population for the VFC programs by tertiles. Panel C shows spatial accessibility by tertile of accessibility score for both rural and urban ZCTAs. Accessibility was defined as supply (ie, VFC provider locations) of and demand for services (ie, children and adolescents age-eligible for the VFC program) within a specified catchment area (ie, 30 minutes’ drive time). Panel D shows spatial accessibility cold spots and hot spots (areas of low access [cold spots] and high access [hot spots]) across ZCTAs. Sources: South Carolina Department of Health and Environmental Control (VFC data, 2019) and the American Community Survey (2013–2017).

## Background

The Advisory Committee on Immunization Practices recommends routine human papillomavirus (HPV) vaccination for male and female adolescents aged 11 or 12 years, beginning as early as age 9, with catch-up vaccination for all people through age 26, and shared clinical decision making before vaccination decisions for those aged 27 to 45 (1,2). Although uptake of HPV vaccination has increased since its initial recommendation (2006 for girls; 2011 for boys), rural populations have lower rates of initiation and completion compared with their urban counterparts, particularly in the southern United States (3). To improve rural HPV vaccination rates, several policy recommendations have been made, including increasing access to the Vaccines for Children (VFC) program through federally qualified health centers, rural health clinics, health departments, and other settings, including pharmacies (4).

The VFC program is a federally funded program that provides vaccines at no cost for certain populations (5). Children through age 18 years who are uninsured, underinsured, Medicaid eligible, or of American Indian/Alaska Native descent can access free HPV vaccination through VFC-enrolled providers.

In South Carolina, rural residents aged 13 to 17 years have lower rates of HPV vaccination initiation (62.2% in 2018) compared with their urban counterparts (79.8% in metropolitan central cities vs 66.8% in metropolitan noncentral cities) (6). To better understand potential drivers of this rural–urban disparity, our objective was to examine spatial access to VFC-enrolled clinics across rural and urban areas of South Carolina.

## Data Sources and Map Logistics

We obtained and geocoded addresses of publicly accessible VFC-enrolled providers from the South Carolina Department of Health and Environmental Control. Nonpublicly accessible VFC providers (eg, juvenile detention centers) were excluded. We also obtained Zip Code Tabulation Area–level (ZCTA, which are geographic approximations of zip codes) population estimates of persons under the age of 18 (ie, age grouping of available data that are most congruent with VFC eligibility criteria) from the 2013–2017 American Community Survey (7). We then performed the 2-step floating catchment area (2SFCA) method in ArcGIS 10.5.1 (Esri) to determine spatial access to VFC providers at the ZCTA–level. The 2SFCA method considers the supply (ie, VFC provider locations) of and demand for services (ie, children and adolescents) within a specified catchment area (ie, 30 minutes’ drive time) to generate a score indicating access to VFC providers for each ZCTA. Thus, for example, a ZCTA may have many VFC providers, but if the 30-minute catchment area has a large population, it will have a smaller access score compared with ZCTAs with fewer providers but a relatively smaller population. Potential values can range from 0 (no access within 30 minutes) to 1 (an improbable 1:1 VFC provider:child ratio). Additional details about this approach are detailed elsewhere (8). We calculated travel distance from the centroid of each ZCTA to the nearest VFC provider, which enables us to determine proximity to VFC providers but does not account for potential demand for services. ZCTAs were categorized as rural or urban using rural–urban commuting area primary codes, with a code of 4 or more categorized as rural (9).

We then performed Optimized Hot Spot Analysis by using the Getis-Ord Gi* tool (10). This statistic identifies where areas of high access (hot spots) and low access (cold spots) are clustered, while adjusting for false discovery rates and spatial dependence. We examined rural–urban differences in VFC providers by type (eg, public health department), spatial accessibility scores, distance to the nearest VFC provider, and hot spots and cold spots by using independent *t* tests and χ^2^ analyses for continuous and categorical variables, respectively.

## Highlights

South Carolina has 493 public VFC providers across rural and urban ZCTAs (panel A). Rural and urban VFC providers varied by type, with the largest proportion of rural providers (41.1%) at federally designated health care centers and the largest proportion of urban providers (55.6%) at private clinics (*P* < .001) (Table 1).

**Table 1:** Vaccines for Children (VFC) Provider Type Across Rural and Urban Designated Zip Code Tabulation Areas (ZCTAs), South Carolina^a^

Provider Type	Rural ZCTA (n = 151), No. (%)	Urban ZCTA (n = 342), No. (%)	*P* Value
Hospitals	10 (6.6)	24 (7.0)	<.001
Private clinics	41 (27.2)	190 (55.6)	
Federally designated health care centers^b^	62 (41.1)	71 (20.8)	
Public health departments	27 (17.9)	28 (8.2)	
Not specified	11 (7.3)	29 (8.5)	

a Analysis using data from the South Carolina Department of Health and Environmental Control (VFC data, 2019) and the American Community Survey (2013–2017).

b Includes federally qualified health centers, rural health clinics, and other community health centers.

Panel B shows the number of children under age 18 within each ZCTA, by tertile. Panel C shows rural and urban ZCTAs by tertiles of access scores. Panel D shows the findings of the Getis-Ord Gi* analysis of these data, indicating hot spots (ie, clusters of high access) primarily toward the southernmost rural tip of the state and toward the northeastern part of the state. Rural ZCTAs had higher mean access scores compared with urban ZCTAs (0.000548 vs 0.000419, *P* < .001) (Table 2). There was no difference in distance to the nearest VFC provider across rural and urban ZCTAs (7.50 vs 6.47 miles, *P* = .06). A higher proportion of rural ZCTAs (16.0%) was in hot spots compared with urban ZCTAs (4.7%) (*P* < .001).

**Table 2:** Spatial Access to Vaccines for Children (VFC) Provider Locations by Rural–Urban Designation Across Zip Code Tabulation Areas, South Carolina

Designation	Rural (n = 125)	Urban (n = 299)	*P* Value
**Clusters type^a^, n (%)**			
Hot spot	20 (16.0)	14 (4.7)	<.001^b^
Cold spot	1 (0.8)	8 (2.7)	
Nonsignificant	104 (83.2)	277 (92.6)	
**Access score^c^, mean (SD)**	0.000548 (0.000357)	0.000419 (0.000294)	<.001^d^
**Distance to the nearest VFC provider, mean (SD), miles**	7.50 (4.92)	6.47 (5.04)	.06^d^

a Analysis using data from the South Carolina Department of Health and Environmental Control (VFC data, 2019) and the American Community Survey (2013–2017). Clusters are identified as areas of high access (hot spots) and low access (cold spots).

b *P* value from χ^2^ test.

c The supply (ie, VFC provider locations) of and demand for services (ie, children and adolescents) within a specified catchment area (ie, 30 minutes’ drive time).

d From independent *t* test.

## Actions

We found that children and adolescents in rural ZCTAs in South Carolina have greater access to VFC providers than those in urban ZCTAs, as demonstrated by higher mean accessibility scores across rural ZCTAs, comparable distances to the nearest VFC provider in urban ZCTAs, and a higher proportion of rural ZCTAs in hot spots. This information suggests that lower HPV vaccination initiation and completion rates in rural South Carolina are likely due to factors other than limited spatial access to VFC providers. Previous studies found lower levels of HPV awareness and knowledge among rural residents compared with urban residents (11). Additionally, providers are less likely to engage in collaborative communication about HPV and HPV vaccination with rural parents (11). Educational interventions, awareness campaigns, and enhanced provider training may be effective ways to improve uptake, especially considering the relatively high availability of VFC programs in many rural areas in the state (12).

Identifying clusters with limited access to VFC providers may help health care systems, public health departments, and policy makers engage in targeted efforts to increase VFC enrollment and thereby expand access to vaccination for vulnerable children and adolescents. Reducing out-of-pocket costs associated with vaccination, implementing vaccination programs in schools and child care centers, and offering vaccinations through home health visits and at pharmacies are also recommended, evidence-based strategies (13,14). Previous studies have shown the utility of using GIS approaches to identify low access areas and target them for additional programs (15).

The Community Preventive Services Task Force has also identified multiple provider-level or systems-level strategies to increase vaccine uptake, such as the use of provider reminder systems and standing orders (16). Understanding the distribution and diversity of VFC providers across rural and urban areas may help inform planning for and delivery of these types of interventions. We found that more than half of rural VFC providers are in federally designated community-based clinics. Community-based clinics are important for expanding access to HPV vaccination for rural populations, and are optimal sites for implementing systems, tools, and protocols that improve vaccination rates (4).
